# Novel *MEIOB* variants cause primary ovarian insufficiency and non-obstructive azoospermia

**DOI:** 10.3389/fgene.2022.936264

**Published:** 2022-08-05

**Authors:** Yurong Wang, Ling Liu, Chen Tan, Guiquan Meng, Lanlan Meng, Hongchuan Nie, Juan Du, Guang-Xiu Lu, Ge Lin, Wen-Bin He, Yue-Qiu Tan

**Affiliations:** ^1^ Hunan Guangxiu Hospital, Hunan Normal University, Changsha, China; ^2^ Institute of Reproductive and Stem Cell Engineering, School of Basic Medical Science, Central South University, Changsha, China; ^3^ National Engineering and Research Center of Human Stem Cells, Changsha, China; ^4^ Reproductive and Genetic Hospital of CITIC-Xiangya, Changsha, China; ^5^ Clinical Research Center for Reproduction and Genetics in Hunan Province, Changsha, China

**Keywords:** primary ovarian insufficiency, non-obstructive azoospermia, MEIOB gene, novel variant, immunofluorescence

## Abstract

**Background:** Infertility is a global health concern. *MEIOB* has been found to be associated with premature ovarian insufficiency (POI) and non-obstructive azoospermia (NOA), but its variants have not been reported in Chinese patients. The aim of this study was to identify the genetic aetiology of POI or NOA in three Han Chinese families.

**Methods:** Whole-exome sequencing (WES) was used to identify candidate pathogenic variants in three consanguineous Chinese infertile families with POI or NOA. Sanger sequencing was performed to validate these variants in the proband of family I and her affected family members. *In vitro* functional analyses were performed to confirm the effects of these variants.

**Results:** Two novel homozygous frameshift variants (c.258_259del and c.1072_1073del) and one novel homozygous nonsense variant (c.814C > T) in the *MEIOB* gene were identified in three consanguineous Han Chinese families. *In vitro* functional analyses revealed that these variants produced truncated proteins and affected their function.

**Conclusion:** We identified three novel *MEIOB* loss-of-function variants in local Chinese patients for the first time and confirmed their pathogenicity using *in vitro* functional analyses. These results extend the mutation spectrum of the *MEIOB* gene and have important significance for genetic counselling in these families.

## Introduction

Infertility is a worldwide health problem, defined as a failure to achieve pregnancy after 12 months of unprotected intercourse (Audibert and Glass, 2015). Infertility can be attributed to female or male factors, or both. The causes of male infertility include oligozoospermia, asthenozoospermia, and azoospermia among others (Agarwal et al., 2021). Non-obstructive azoospermia (NOA) is a serious male infertility disorder that accounts for more than 20% of the male infertility issues (Cioppi et al., 2021). NOA is typically associated with genetic defects, including Y chromosome microdeletions and spermatogenesis related gene mutations (TEX11, HFM1, and MEI1) (Yatsenko et al., 2015; Ben Khelifa et al., 2018; Tang et al., 2021). The causes of female infertility include premature ovarian insufficiency (POI), abnormal ovulation, tubal blockage and endometriosis (Sen et al., 2014). POI is characterized by loss of normal ovarian function in women before 40 years of age (De Vos et al., 2010). It has been reported that a number of genes are responsible for POI, such as DNA damage repair relevant genes (FANCA, DMC1, MCM8, etc) (Desai et al., 2017; He et al., 2018; Yang et al., 2019) and hormone receptor genes (FSHR, LHCGR, etc) (He et al., 2019; Guo et al., 2021; Tang et al., 2021).

Meiosis is an important process in gametogenesis, and defects in several meiosis-related genes have been reported in relation to POI and NOA (*MEI1*, *DMC1*, and *SPO11* (Zhang et al., 2020; Verrilli et al., 2021). *MEIOB* is a meiosis-specific gene mainly involved in double-strand break (DSB) repair, crossover formation, and promotion of complete synapsis (Luo et al., 2013). *MEIOB*-knockout mice presented with meiotic arrest, resulting in female and male infertility (Caburet et al., 2019). However, variants of *MEIOB* associated with human infertility are rare, with only 10 cases reported so far (Table 1), and none of the variants have been reported in the Chinese patients with POI or NOA (Gershoni et al., 2017; Caburet et al., 2019; Gershoni et al., 2019; Krausz et al., 2020; Cannarella et al., 2021; Wu et al., 2021; Kherraf et al., 2022).

**TABLE 1:** The currently reported phenotypes and genotypes of *MEIOB* gene in infertility patients.

NO.	cDNA change	Amino acid change (region)	Phenotype of subjects	References
1	c.191A > T (hom)	p.N64I(OBCD1)	NOA	Gershoni et al. (2017)
2	c.1098del (hom)	p.S366fs (OBCD3)	NOA	Gershoni et al. (2019)
3	c.1218G > A (hom)	p.Cys345Trpfs8(OBCD3)	POI	Caburet et al. (2019)
4	c.897_1431del (hom)	p.219del178aa (OBCD2)	NOA	Krausz et al. (2020)
5	c.1140_1143del (hom)	p.Cys345Trpfs8(OBCD3)	NOA	
6	c.318C > A (het)	p.Ser106Arg (OBCD1)	oligozoospermia	Cannarella et al. (2021)
7	c.634G > A (het)	p.Asp212Asn(OBCD2)	oligozoospermia	
	c.643 T > G (het)	p.Ser215Ala (OBCD2)		
8	c.634G > A (het)	p.Asp212Asn(OBCD2)	NOA	
	c.* 4G > A (het)			
9	c.683-1G > A (hom)		NOA and POI	Wu et al. (2021)
10	c.1118-1121del (hom)	p.phe373ser (OBCD3)	NOA	Kherraf et al. (2022)

In this study, we recruited three consanguineous Chinese infertile families with POI or NOA. Three novel *MEIOB* variants were identified using whole-exome sequencing (WES). These mutations have been confirmed as pathogenic variants using bioinformatics and *in vitro* functional analyses, thereby broadening the MEIOB gene mutation spectrum.

## Materials and methods

### Study subjects

Three individuals from three consanguineous Han Chinese families were recruited from the Reproductive and Genetic Hospital of CITIC-Xiangya, after providing informed written consent. Two female probands were diagnosed with POI and one male proband was diagnosed with NOA. Diagnostic Criteria of POI refers to the depletion or loss of normal ovarian function in women before 40 years of age, whose clinical characteristics are sparse menstruation or amenorrhea >4 months, and two times of the circulating gonadotropin follicle-stimulating hormone (FSH; >25 mIU/ml) elevated (interval of more than 4 weeks) (De Vos et al., 2010). The Diagnostic Criteria of NOA refers to azoospermia of semen routine examination that after excluding obstruction and hormone factors, and No karyotype abnormality and AZF microdeletion. Subsequently, molecular diagnosis was confirmed through WES (Minhas et al., 2021). This study was reviewed and approved by the ethics committee of the Reproductive and Gxenetic Hospital of CITIC-Xiangya of Central South University, China.

### Genomic DNA extraction and WES

Genomic DNA from peripheral blood samples was extracted using a QIAamp^®^ DNA Blood Midi Kit (Qiagen, Germany), according to the manufacturer’s protocol. All probands were subjected to WES, which was performed using the HiSeq2000 sequencing platform (Illumina). The WES raw reads were aligned to NCBI GRCh37 (reference genome Hg19) using Burrows-Wheeler Aligner, while Picard was used to remove and sort the copies of the polymerase chain reaction (PCR; http://broadinstitute. gib. io/picard/). The GATK package was used for mutation identification, including base recalibration variant calling with haplotype caller and variant quality score recalibration, and ANNOVAR software was used for mutation annotation.

**FIGURE 1:**
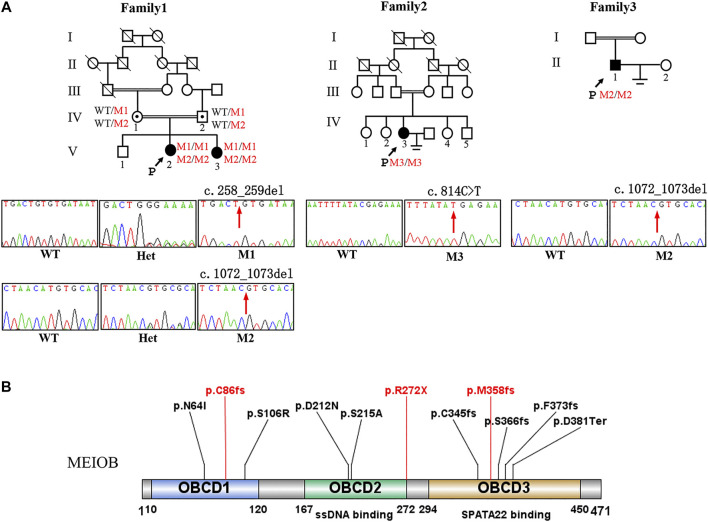
Pedigree of three consanguineous Han Chinese family with infertility-related MEIOB variants. **(A)** The black arrow points to the proband. Open symbols indicate the unaffected members. Heterozygous carriers are indicated with a dot in the middle of the symbol. Filled symbols indicate the affected members with POI or NOA. In familyⅠ, two cousins (Ⅳ-1 and Ⅳ-2) in generation 4 married to each other with two affected individuals (V-2 and V-3 with POI). Sanger sequencing of the c.258_259del and c.1072_1073del (pointed by red arrows) mutation of *MEIOB* in this family. **(B)** The MEIOB protein consists of 471 amino acids, including three OB- domains. Three variants discovered in this study are shown in red font, whereas previously reported variants are displayed in black font.

We used the following inclusion criteria to identify candidate genes: 1) Frequencies are less than 1% in database of 1,000 Genomes, gnomAD, gnomAD-EAS; 2) Homozygous variants are preferred considered in consanguineous family; 3) The potential pathogenicity of the novel variants were examined by in silico analysis using two different software: Mutation Taster and CADD; 4) Candidate genes which are recorded in OMIM database and responsible for POI or NOA; 5) Candidate genes which are related to POI or NOA in animal models. The suspected variants were validated by Sanger sequencing of the probands and their family members (Figure 1A), and the primers are listed in Supplementary Table S1.

### Plasmid construction

Full-length *MEIOB* was obtained by PCR and inserted into the pcDNA3.1/FLAG expression vector, as described previously, leading to the production of fusion proteins with FLAG at the N-terminus of *MEIOB* (*MEIOB*-WT). The identified mutations (c.258_259del,c.1072_1073del, and c. C814T) were introduced into the *MEIOB*-WT plasmid vector by oligonucleotide-mediated mutagenesis using a MutExpress II Fast Mutagenesis Kit (Vazyme, Guangzhou, China). All expression constructs were sequenced to confirm the presence of the desired mutation and to exclude PCR-induced mutations.

### Western blot analysis and immunofluorescence analysis

Cells were harvested 48 h after transfection and homogenised using RIPA lysis buffer (Beyotime Biotechnology, Shanghai, China) supplemented with a protease inhibitor cocktail (Thermo Fisher Scientific, United States). Proteins extracted from the transfected cells were blotted onto a polyvinylidene difluoride membrane and incubated overnight at 4°C with an anti-FLAG antibody (1:5,000 dilution, Abways, Shanghai, China). The membrane was incubated with secondary antibodies (goat anti-mouse IgG; 1:5,000 dilution; Abways, Shanghai, China) on the following day. Finally, blots were developed using an ECL western blotting kit (Pierce Biotechnology, Rockford, IL, United States).

Immunofluorescence analysis was performed on transfected Chinese hamster ovary (CHO) cells grown on coverslips using anti-FLAG (1:500 dilution) primary antibodies. Fluorescent images were captured using a confocal microscope (Olympus FV1000, Tokyo, Japan).

## Results

### Phenotype of the patients with the MEIOB variant

In the first family, the proband (Ⅴ3, Figure 1A) had started menstruating at the age of 15 years with normal periods. She was infertile for 2 years after marriage and had normal sexual intercourse without contraception. She was diagnosed with primary infertility at the age of 28 years. The clinical manifestations included oligomenorrhea and infertility. Her sex hormone levels showed an elevated FSH level (38.44 IU/L) and a decrease in anti-Mullerian hormone (AMH) levels (0.098 ng/ml). Transvaginal ultrasound revealed that the uterus was of normal size, the left ovary had a small follicle, while no follicles were detected in the right ovary (Supplementary Figure S1). The proband’s parents and grandparents were consanguineous. The 29-year-old sister of the proband was also diagnosed with POI, with elevated FSH and decreased AMH (0.4 ng/ml) levels.

The proband (Ⅳ3, Figure 1A) of the other family (family II), was a 24-year-old woman, and was diagnosed with POI. She had been infertile for 2 years after marriage following normal sexual intercourse without contraception. Her parents were consanguineous. She had normal pubertal development and a regular menstrual cycle. Transvaginal ultrasound revealed follicles only one follicle in each ovary (Supplementary Figure S1). Basal endocrine testing showed an elevated FSH level of 29.88 IU/L and a low AMH level of 0.092 ng/ml.

The proband (II2) of the third family (family III) was a 29-year-old man and suffering from primary infertility. The patient was diagnosed with non-obstructive azoospermia (NOA) with a normal volume of semen analyses. Endocrine tests revealed normal hormone levels. The proband had a normal 46, XY karyotype, and no abnormalities were observed in the Y chromosome microdeletion detection.

The spouses of our two POI patients have a normal karyotype (46, XY), and have no anomalies in Y chromosomal microdeletion detection or in semen routine examination. The NOA patient’s spouse has normal menstrual cycle and sex hormone levels. Transvaginal ultrasound showed she has normal inner reproductive organ (oviduct, ovary and uterine) and she had a good clinical pregnancy after undergoing IVF-ET with donor sperm.

### Identification of *MEIOB* variant

WES was performed on the three probands. According to the filtering criteria, two novel homozygous frameshift variants of *MEIOB* (NM_001163560: c.258_259del: p. C86fs; c.1072_1073del: p.M358fs) were identified in the proband of family I. The subsequent Sanger sequencing showed that her affected sister carried the same homozygous variants and her parents both were heterozygous variants carriers. In addition, a novel homozygous nonsense variant and a novel homozygous frameshift variant (NM_001163560: c.C814T:*p*.R272X; c.1072_1073del: *p*.M358fs) were detected in the probands of family II and III, respectively. According to the ACMG standards and guidelines (Richards et al., 2015), all the detected candidate variants were evaluated as pathogenic (c.258_259del: PVS1+PM2+ PM3_Supporting + PP1; c.1072_1073del: PVS1+PM2+PM3_Supporting + PP1; c. C814T: PVS1+PM2+PM3_Supporting).

### Impact of MEIOB variant

**FIGURE 2:**
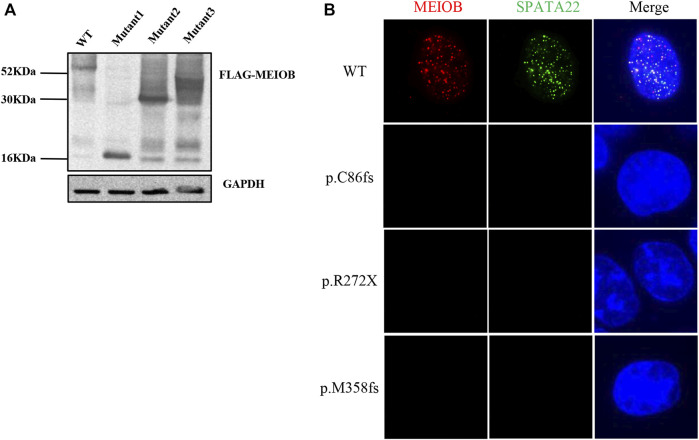
*In vitro* functional analysis of MEIOB variant. **(A)** Western blot analysis showed that the three mutant proteins were expressed in transfected cells, which resulted in the production of truncated proteins. **(B)** Immunofluorescence analysis showed that the wild-type MEIOB colocalised with SPATA22 to ssDNA; the truncated MEIOB was not located in the nucleus.

Western blot analysis showed that the three mutant proteins were expressed in human embryonic kidney (HEK293T) cells after transfection with the expression construct and production of truncated proteins. Immunofluorescence analysis showed that the wild-type MEIOB colocalised with SPATA22 to ssDNA; however, MEIOB was not located in the nucleus when cells were transfected with the mutant constructs. *In vitro* functional analyses revealed that all these variants lost the function of MEIOB (Figure 2).

## Discussion

In this study, we performed WES to identify two novel homozygous frameshift mutations (c.258_259del and c.1072_1073del) and one novel homozygous nonsense mutation (c.814C > T) in the *MEIOB* gene from three consanguineous Han Chinese families. *In vitro* functional analyses revealed that these mutations produce truncated proteins and affect their function, which might result in the impairment of chromosomal break repair and ultimately lead to male and female infertility. Our study broadens the mutation spectrum of *MEIOB*.

The *MEIOB* gene is located on chromosome 16, consists of 14 exons, and encodes 471 amino acids. It is a meiosis-specific ssDNA-binding protein that participates in homologous recombination and DSB repair (Souquet et al., 2013). *MEIOB* knockout mice (*Meiob* ^*−/−*^) exhibit infertility in both sexes, resulting from meiotic arrest at a zygotene/pachytene-like stage (Luo et al., 2013). Previous studies have reported that mutations in MEIOB may cause infertility in males and females (Gershoni et al., 2017; Caburet et al., 2019). However, only 10 pathogenic MEIOB mutations have been described to date. In our study, three novel MEIOB mutations were identified in patients with POI or NOA. *In vitro* functional analyses showed that these variants lost their functions. Therefore, we suggest that the three *MEIOB* mutations are deleterious and disease-associated mutations in the three families.

The MEIOB protein contains three conserved OB binding domains (OBCD), of which OBCD2 (167-272aa) is responsible for binding the DNA single strand to RPA and OBCD3 (294-450aa) is the interaction domain with SPATA22 (Ribeiro et al., 2016) (Figure 1B). It has been reported that MEIOB first forms the dimerisation core of MEIOB-SPATA22 and subsequently interacts with the preformed RPA complex to participate in meiotic homologous recombination (Ribeiro et al., 2021). Previous studies have shown that the stability of these two proteins (MEIOB and SPATA22) is determined by their interaction (Xu et al., 2017), which is essential for meiosis and gametogenesis. The SPATA22 defect leads to gamete arrest during the pachytene phase of the first meiotic division both in human and mice, consequently the sterile phenotype of POI and NOA (Hays et al., 2017; Yao et al., 2022). In our study, the three novel mutations of MEIOB were located in each of the three OB-binding domains and the production of truncated proteins without OBCD3. Furthermore, immunofluorescence analysis revealed that mutant MEIOB proteins did not co-localise with SPATA22 in the nucleus. Therefore, we suggest that OBCD3 in MEIOB is important for human fertility.

Different types of variants might have distinguishable impacts on protein function and subsequently lead to different clinical phenotypes (Spillane et al., 2016). In previous studies, it was found that nonsense and frameshift variants of *MEIOB* might both have a greater impact on protein function, resulting in a more severe clinical phenotype. For example, the first homozygous nonsense variant (c.1218G > A) in *MEIOB* causes POI, whereas the compound heterozygous missense variants (c.634G > A and c.643T > G) lead to oligozoospermia instead of NOA (Caburet et al., 2019; Cannarella et al., 2021). In our study, the two novel homozygous frameshift variants (c.258_259del, c.1072_1073del) and one novel homozygous nonsense variant (c.814C > T) in the *MEIOB* gene both caused POI or NOA. Therefore, we suggest that loss-of-function variants of *MEIOB* result in a severe clinical phenotype such as POI or NOA.

It has been reported that mutations in DSB repair-related genes might increase the risk of cancer. For example, mutations in *BRCA1* and *BRCA2* increase the risk of breast cancer, and mutations in *RAD51* increase the risk of rectal cancer (Elstrodt et al., 2006; Osti et al., 2017). *MEIOB* was recently identified as a new cancer testis gene (Wang et al., 2016). Overexpression of the *MEIOB* gene has been observed in triple-negative breast cancer (TNBC) cells, which is confirmed that ectopic expression of *MEIOB* is oncogenic and promotes cancer cell proliferation (Gu et al., 2021). In our study, the patients did not present with any cancer. However, we recommend that patients who have mutations in the *MEIOB* or other DSB repair-related genes, should undergo regular check-up and screening for early detection and timely intervention of cancer.

In conclusion, we identified three novel *MEIOB* loss-of-function mutations in Chinese patients for the first time and confirmed their pathogenicity using *in vitro* functional analyses. These results extend the mutation spectrum of the *MEIOB* gene and have important significance for genetic counselling of families with infertility.

## Data Availability

The raw data supporting the conclusions of this manual will be made available by the authors, without under reservation, to any qualified researcher.
